# Principles and Framework for eHealth Strategy Development

**DOI:** 10.2196/jmir.2250

**Published:** 2013-07-30

**Authors:** Richard E Scott, Maurice Mars

**Affiliations:** ^1^Nelson R Mandela School of MedicineDepartment of TeleHealthUniversity of KwaZulu-NatalDurbanSouth Africa; ^2^Office of Global e-Health and StrategyFaculty of MedicineUniversity of CalgaryCalgary, ABCanada

**Keywords:** eHealth strategy, eHealth strategy development framework, eHealth, telehealth, telemedicine, e-learning

## Abstract

Significant investment in eHealth solutions is being made in nearly every country of the world. How do we know that these investments and the foregone opportunity costs are the correct ones? Absent, poor, or vague eHealth strategy is a significant barrier to effective investment in, and implementation of, sustainable eHealth solutions and establishment of an eHealth favorable policy environment. Strategy is the driving force, the first essential ingredient, that can place countries in charge of their own eHealth destiny and inform them of the policy necessary to achieve it. In the last 2 years, there has been renewed interest in eHealth strategy from the World Health Organization (WHO), International Telecommunications Union (ITU), Pan American Health Organization (PAHO), the African Union, and the Commonwealth; yet overall, the literature lacks clear guidance to inform countries why and how to develop their own complementary but locally specific eHealth strategy. To address this gap, this paper further develops an eHealth Strategy Development Framework, basing it upon a conceptual framework and relevant theories of strategy and complex system analysis available from the literature. We present here the rationale, theories, and final eHealth strategy development framework by which a systematic and methodical approach can be applied by institutions, subnational regions, and countries to create holistic, needs- and evidence-based, and defensible eHealth strategy and to ensure wise investment in eHealth.

## Introduction

Many definitions of eHealth have been developed or adopted, but perhaps the bottom-line message is that eHealth can be anything we want it to be. It is simply the application of information and communications technologies (ICTs) to the health sector [1]. Evidence shows eHealth is now a globally pervasive tool [2] yet seldom have health organizations, countries, or geographic regions had a proper eHealth strategy to guide implementation. Why then the renewed discussion about “eHealth strategy” in developing countries and regions during 2011? For example, the Pan American Health Organization (PAHO) promulgated its Regional eHealth Strategy approach at their 51^st^ Directing Council meeting [3], and in February 2011 the African Union resurrected past debate around the issue at an Experts Meeting on eHealth and Telemedicine Harmonization in Africa. Similarly, Kenya just completed a 2-year undertaking to develop its eHealth strategy [4], and South Africa has just released its revised eHealth Strategy [5]. In 2012, a WHO/ITU collaboration released its WHO-ITU National eHealth Strategy Toolkit [6]. Perhaps the need is finally being understood. However, although these documents provide some insight, specific guidance for individual countries or institutions to design and develop their own eHealth strategy is unclear and is lacking in the literature.

As a consequence, entities will often emulate or adapt practice from elsewhere. While emulation or adaptation is common, these approaches are inappropriate: “emulation” because solutions and approaches must be context specific, and “adaptation” because, although a compromise, it remains suboptimal. A sustainable eHealth solution is best designed and developed organically and interactively with stakeholders within the context and setting in which it will be applied, and in alignment with the existing health, education, and technology enterprises.

According to Mintzberg and Lampel [7], the strategy literature began to unfold in the 1960s. Use of “strategy” development, once commonly applied by the private sector, has faded. Within the eHealth arena, high-level policy statements and “road-maps” are sometimes referred to as “strategy” but do not provide the evidence base and structure desirable for sustainable eHealth implementation. This current paper questions the value of eHealth for developing countries, demonstrates the need for eHealth strategy, and identifies three available tools, before enhancing one of these tools by embedding within it recognized strategy concepts and cognitive assessment approaches to create an enhanced eHealth Strategy Development Framework.

Growing expectations, changing demographics, and resource limitations require wise investment in eHealth solutions that address major health needs. Of even greater import, eHealth activities implemented now will establish the practice and technology infrastructure for decades to come. Sustainable eHealth solutions require development of a sound, evidence-based, and defensible eHealth strategy. Application of the enhanced eHealth strategy development tool presented here is recommended as a key initial step and presents health care institutions, subnational regions, or countries with a viable model.

## eHealth Strategy in the Political and Policy Context

A desire exists to believe policy making is rational and based upon best available empirical evidence. Marmot [8] noted that the “evidence-based” movement attempted to influence the political/policy context to create more of an “evidence-based policy making” process, as opposed to making the evidence fit the political/policy context (termed “policy-based evidence making”). Within that frame, a very linear process was perceived: A policy issue would be identified, the scientist would gather the evidence, KT (knowledge transfer/translation) would ensure the evidence got to those who needed it, and decision-makers would inevitably make evidence-based decisions. However, examples suggest that policy making is not, in fact, based upon such a linear process or on the best available empirical evidence [9,10]; rather, it is often based on public opinion, electoral considerations, personal preference, and crisis management.

Some authors have taken a cautious, even cynical, view of evidence-based policy making and practice while others, however, point more optimistically to recent changes in attitude. For example, Fafard [11] states that just as evidence-based medicine requires systematic analysis of available evidence, so too should evidence-based public policy be based on the careful testing of different policy and program options and notes that this is where the role of empirical evidence is the strongest. The author then concludes that two significant changes have occurred; first, there has been a shift from “evidence-based” to “evidence-informed” policy making, and second, there is renewed interest in taking into account the real life *context* of decision making.

It would seem that careful research is still required to make choices between an array of possible policy instruments and program interventions. This is particularly so in complex fields such as health, health care, and eHealth. The approach described in this paper ensures the evidence is provided and current context is thoroughly understood (including underlying values and value conflicts), and it therefore supports evidence-*informed* decision making regarding possible application of eHealth solutions.

## Available Guidance for eHealth Strategy Development

Many developed countries (eg, Australia, EU countries) have established a variety of documents termed, or akin to, “eHealth strategy” [12,13]. They provide examples, but little or no guidance to the process of development. Furthermore, as described above, emulation or adaptation of approaches from elsewhere is not recommended. Recently Jones [14] published a strategy development guide that was specifically eHealth focused. While providing useful tools and guidance, it lacks theory and a holistic approach. In late 2012, the World Health Organization (WHO) and International Telecommunications Union (ITU) released their WHO-ITU National eHealth Strategy Toolkit [6], intended to provide a strategic framework and method for the development of a national eHealth vision, action plan, and monitoring and evaluation framework. This also provides useful tools and guidance, but its comprehensiveness may lead to complexity in its execution. Scott [15] first provided a framework for Strategic Planning in relation to eHealth, and it is that framework that provides systematic process, direction, and coherence, allowing any entity—regional, national, subnational, or facility—to develop its own eHealth strategy, leading to significant and measureable future impact.

## Need for and Value of Developing an eHealth Specific Strategy

### Is There a Need for eHealth Strategy Development?

eHealth in its largest sense has been practiced for many decades now—from basic telephony, through transmission of ECGs and images, to comprehensive e-records and even remote surgery. But despite this experience, there are few sustained eHealth implementations of demonstrated success and sustainability as evidenced through rigorous evaluation. The ITU [16] stated that, for at least the period 1960-2000, the “traditional cycle of telemedicine projects” was disappointing, and they noted that thousands of pilot sites, trials, tests, etc, took place but few of the initiatives survived beyond the end of their initial funding period. They concluded that, during the 20th century, perhaps fewer than 10% of projects in developing countries were successful, with 45% faltering after just 1 year and the remaining 45% after 3 years. There is little reason to believe this has changed for initiatives implemented in the new century. Indeed, Ekeland et al [17] commented that available evidence on the value of telemedicine varies from “promising but incomplete” to “limited and inconsistent”, with a particularly problematic area being economic analysis of telemedicine. Similarly, van Eland-de Kok et al [18] identified only small to moderate positive effects of eHealth on primary health outcomes of chronic disease patients and noted that due to the limited number of studies and methodological limitations, the evidence was not fully convincing.

A similar circumstance exists for large-scale electronic record initiatives, with large health informatics applications in developed countries failing to prove as successful as desired. For example, Electronic Health Records (EHRs) have been, or continue to be, introduced in many developed countries such as England, Scotland, France, Canada, Australia, and the USA—and at significant cost and risk. Originally budgeted at £2.3 billion, the United Kingdom is estimated to have spent between £6.2 billion [19] and £20 billion [20] on its NHS Connecting for Health program—and abandoned the program in 2011 as largely a failure [21]. Some estimates of Canada’s pan-Canadian eHealth initiatives suggest a total expenditure of $10 billion [22] (with additional investment by provinces and territories), and questions of value have arisen in Ontario, British Columbia, and Alberta [23]. The bulk of these expenditures have been borne by the public sector, given that the private sector avoids investment until it sees a sound market opportunity. Black et al [24] completed a “systematic review of systematic reviews” of various eHealth solutions on the quality and safety of care and concluded that “despite support from policy makers, there was relatively little empirical evidence to substantiate many of the claims made in relation to these technologies”. Also, Jamal et al [25] systematically reviewed the impact of health information technology (HIT) or health information systems (HISs) on the quality of health care and found insufficient evidence of either clinically or statistically important improvements in patient outcomes.

In regard to developing countries, Fernandez and Oveido [26] observed that, for the Caribbean region, it is only well-managed health institutions that plan medium- and long-term eHealth programs that are likely to be able to implement successful initiatives. According to these authors, ICT projects in the region are usually short-term and unsustainable, due to expectations of “instant results” and a lack of support for the new projects stemming from a lack of knowledge and understanding by policy and decision makers. They also highlight the lack of standardization needed to encourage the interjurisdictional sharing of information. These observations are likely to be equally applicable to institutions, as well as health systems, in most other developing countries and regions.

It would seem clear that our current approach to eHealth implementation does not work, and an alternate approach is needed.

### Is eHealth a Viable Solution for Developing Countries?

The potential of eHealth to address growing health system concerns and health care needs is often identified in the literature, but clear evidence of its value remains uncertain. With these perspectives in mind, it is reasonable to ask “is eHealth a viable solution for developing countries?”

Despite the lack of success described above, there is some evidence from the developed world that HISs address health concerns and may lead to cost savings. But, even then, are the health concerns addressed by HISs in developed countries (eg, reduced adverse drug reactions) the most relevant to the developing world? Furthermore, the European Commission [27] found that for EHRs and ePrescribing in European countries, at least 4 years (more typically up to 9 years) are required to show positive annual socioeconomic return (SER), and 6-11 years to realize a cumulative net benefit. Given this time to realize SER in developed countries, can developing countries run the risk? Finally, are the projected cost savings for developed countries even feasible elsewhere? The United States spent an estimated US $8650 per capita (almost 18% GDP) on health in 2011, and Canada spent Can $5800 (projected) per capita (11.6% GDP) in 2011 [28]. In health systems that spend $6000 to $9000 per capita on health per year, perhaps there is room for savings through greater efficiencies. But in health systems that spend $10-35 per capita per year (as in many developing countries), are *any* cost savings likely? The business case is unlikely to be made through cost savings alone.

### How Much Is Available to Spend on eHealth Solutions?

The WHO’s Report of the Commission on Macroeconomics and Health [29] identified that countries needed to spend, at that time, a minimum of $34 per capita to provide just a basic health care package to their population. Introducing another element that requires funding, ie, eHealth, becomes an “opportunity cost”. If you spend money on eHealth, you have to take it away from something else—immunization, sanitation, clean water, rural clinics, health provider salaries. Not all the funding will come from donors—sustainable solutions require investment by the country too. So how much does a developing country have available to spend on eHealth? To place this in perspective, consider the following. Of the Can $5800 per capita spent in 2011 by Canada, about 72% (OECD country average) or $4176 came from the public purse. Of this money, nearly 2.7% was spent on technology use in health (only some of which was eHealth), meaning Canada spends around Can $113 per capita on ICT use in health. In a country that spends $10-35 per capita on all its health needs, 2.7% would amount to 27-67 cents per capita on all technology applications. What eHealth solution can be bought, implemented, maintained, and sustained for that price?

### What Is the Value of an eHealth Strategy?

So, is eHealth a solution for developing countries? Perhaps, but the solutions and approaches are unlikely to be those pursued in developed countries and must be aligned with the specific health system and health needs of the entity (institution, subnational region, country) and culture involved [30]. To achieve this very complex goal, an eHealth strategy is essential to provide evidence-based guidance, describe the needs, and justify any expenditure, and thereby ensure wise investment of already incredibly scarce resources.

**Figure 1:**
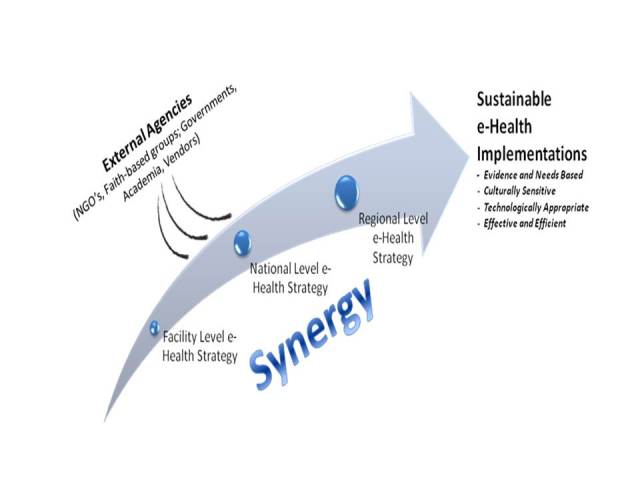
Synergistic benefits derived from an eHealth strategy.

There are also synergistic effects. Once a national eHealth strategy is in place, it encourages (perhaps requires) facility-level eHealth strategy development, which aligns with and supports the national-level approach. Similarly, within a geographic or trading “region”, countries can align their own approaches to develop a regional eHealth strategy. Several benefits are inherent in such an approach. Countries and regions take ownership of their own eHealth destiny and can guide (or decline) opportunities presented by external agencies. Furthermore, the shared experience allows more rapid accomplishment of sustainable eHealth implementations (see Figure 1).

## Invoking “Strategy” and “Complex System Analysis”

### Strategy

Originally a military concept, strategy development became common in the private/business sector during the 1950s and 1960s. Since that time, overt strategy development has faded; some reasons cited include failure to differentiate and treat separately “direction-giving/leadership” and “managing”, “irrational exuberance” of the markets, lack of respect for “direction-giving/leadership” as a profession, and failure to take seriously the need for “strategic thinking” and subsequent implementation in any learning organization [31]. Strategy development in the health sector (particularly for eHealth) is uncommon, yet much could be gained by recognizing the value of strategy development and its application to the eHealth environment.

Strategy in its simplest sense can be considered clarity around *where* you are going and *why* you are going there. According to Porter [32], strategy is creating fit among an organization’s activities (without fit, there is no distinctive strategy and little sustainability), and the success of a strategy depends upon doing many things well and integrating them correctly. In the context of eHealth, an eHealth strategy would be documentation that describes the overall approach to be taken by an entity (institution, subnational region, country). It will identify and implement technologically appropriate and culturally sensitive eHealth solutions in the most appropriate manner and for the most appropriate purposes, explaining not just what is to be done, but why (given the prevailing circumstances). Strategy is key to sustainable eHealth implementation—indeed, the foundation for sustainability is strategy development. Many countries and organizations may claim to have an eHealth strategy (eg, the “Road Maps” of EU countries), but these tend either to be too narrow in focus or too general and abstract and often begin with a goal or an objective that is stated without substantive context and perspective as to its rationale or origin, its impact on prevalent health needs, or any insight around its selection versus alternatives.

**Figure 2:**
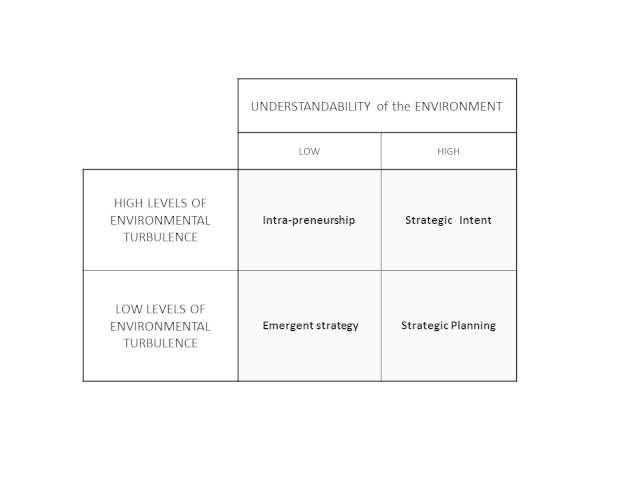
Boisot’s typology of strategy options.

What approach to strategy development is most appropriate for the complexity and continuously developing eHealth setting? Boisot [33] has presented a typology that describes four different kinds of approach to strategy determined by the level of “turbulence” and “understandability” of the setting (Figure 2). According to Boisot, *intrapreneurship* is a state of great unpredictability and flux where entities respond as best they can under the chaotic circumstances surrounding them; *emergent strategy* is the product of “top down” and “bottom up” approaches which emerge incrementally over time without focussed effort; and *strategic intent* is an intuitively clear direction that can be pursued despite the turbulence present and that permits activities to be aligned with a common purpose. Finally, *strategic planning* is viewed as formal consideration of a future course and has value in forcing consideration of two primary factors—the country’s setting, and the inherent uncertainty surrounding eHealth. In this way, the strategic planning process matches appropriate activities to the evolving eHealth environment.

eHealth is recognized to be a constantly evolving field, but the turbulence that existed in the early days has passed. Similarly, sufficient research and application has taken place that sound lessons and good “understandability” exists of where and how to apply eHealth. Thus, within Boisot’s typology, “strategic planning” lies at the intersection of high understandability and low environmental turbulence and is the appropriate strategic option to pursue.

### Complex System Analysis

What makes a setting complex? Often it is the presence of a large number of interconnected parts whose interaction is not merely additive (a “simple” setting), but synergistic where the combinations and permutations are large and the outcomes not always obvious considering the properties of individual components. Complexity can be disorganized or organized [34]: disorganized complexity arises merely through the presence of a very large number of component and interconnected parts; organized complexity arises because the interconnected parts exhibit emergent properties—complex patterns arising out of a multiplicity of relatively simple interactions between even a small number of parts. Human economies, social structures, health systems, and ICT infrastructures are all considered complex settings. It follows therefore that eHealth—with a large and growing number of potential applications (eg, technological and medical/health options), each of which interact with one another creating a complex setting—is certainly complex.

How can such complex settings be assessed? Traditionally, we strive to reduce complex systems to simpler subparts and analyze those. However, in doing so, it can be argued that the “real-life” context and relevance of any analysis is lost. Other approaches are required. One approach is to examine settings in a holistic manner, which (according to the Oxford dictionary) is “characterized by comprehending the parts of something as intimately interconnected and explicable only by reference to the whole”. Holistic analysis is typically interdisciplinary, concerned with the behavior of complex systems and respects occurrence of “feedback” (ie, when information about an event in the past will influence an occurrence (or occurrences) of that same or related event in the present or future).

**Figure 3:**
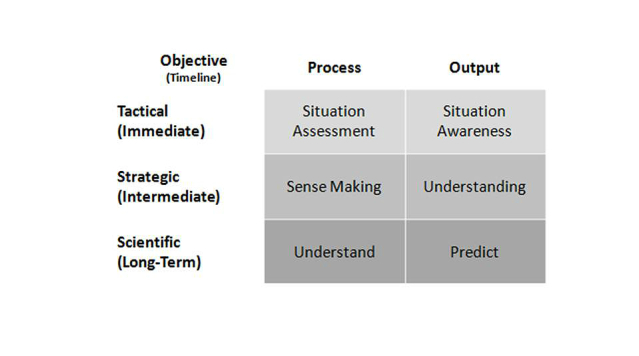
Relationship among several cognitive processes.

Another approach is to mimic something we do innately (eg, when driving) to understand complex and dynamic settings, that is, create “situation awareness” (defined as “the perception of elements in the environment within a volume of time and space, the comprehension of their meaning, and the projection of their status in the near future” [35]). Situation awareness arises when elements within the immediate setting are clearly perceived with respect to time and/or space, their meaning is comprehended, and projections are made of their status within the setting after some variable has changed (eg, time, speed, direction). It is an accepted tool for critical decision making in complex, dynamic areas [36], since current awareness determines what issue(s) are addressed next as well as interpretation of the information perceived [37]. The process by which this is done is termed “situation assessment” (sometimes “situational assessment”) and is a form of tactical analysis that can be related to strategic and scientific analysis as seen in Figure 3.

Combining the approaches of holistic review and situational assessment, performance of a “holistic situation assessment” is recommended and is embedded within the enhanced eHealth strategy development framework described below.

## Principles of eHealth Strategy Development

Before considering development of institutional, regional, or national eHealth strategy and policy, there are some fundamental principles that need be adopted. These are outlined below.

### Principle 1: Simplify Complex Contexts

Experience gleaned from the literature shows the process of integrating eHealth as a routine health care tool faces many challenges, is very complex, and requires significant time. However, by establishing a sound and evidence-based eHealth strategy, it is possible to reduce the impact of such realities. The process is most effective when undertaken by a local (institutional, regional, country) team, as it builds local capacity, is designed by those most intimately knowledgeable about the setting, and establishes pride and commitment of ownership for the undertaking and product.

### Principle 2: A Pragmatic Approach Is Best

The goal of the strategy is to find an optimal solution to the most pressing (existing or anticipated) health-related problems. In other words; the approach is very focused, very health or health care “needs-based”, and strongly “evidence-informed”, but not overly researched (see Step 1 in the Process section below). This requires an understanding of pressing health care needs and alignment with, or creation of, a clear eHealth strategy to address them.

### Principle 3: Spread the Cost

Networking provides opportunities to spread the cost of infrastructure and “infostructure” development between the government, business, agriculture, education, and health sectors. For example, the ICT network supports all these sectors, not just one, and therefore the cost burden should not be borne by just one sector.

### Principle 4: Balance Which eHealth Components Are Applied

Four primary components of eHealth exist [38]: (1) health informatics (collection, analysis, and distribution of health related data; eg, electronic records, surveillance), (2) telehealth (direct or indirect interaction with other health care providers, ill patients, or well citizens, eg, teleconsultation; social networking), (3) e-learning (use of ICT to provide teaching and education opportunities to health care providers and citizens), and (4) e-commerce (related to the business side of health care, eg, electronic reimbursement).

Solutions to specific health issues may require a predominance of one component over others, but it is likely any sustainable and comprehensive solution will require elements of each.

### Principle 5: eHealth Solutions Must Be Right for the Setting

eHealth solutions that are identified for implementation should be technologically appropriate and culturally sensitive.

Appropriate technology can be defined as the most benign technological solution that achieves the desired purpose within the confines of current social, cultural, environmental, and economic conditions of the setting in which it is to be applied and that promotes self-sufficiency on the part of those using it in that setting. Described in this fashion, an appropriate technology would typically be simple to adopt and require fewer resources to operate and maintain (making it more likely to be sustainable and environmentally friendly).

Cultural sensitivity requires solutions to respect local traditions, expectations of the health care system, beliefs about health and disease, and patterns of usage of available health care services. Ignoring local health culture, such as traditional medicines or influential shamans, may undermine efforts to introduce eHealth initiatives. Or insufficient local resources may lead to abuse of modern medicines, such as using reduced doses of antibiotics, which may permit development of resistant strains capable of global spread. Solid experience and knowledge of cultural limitations must guide the design and implementation of eHealth solutions [39].

### Principle 6: Provide Long-Term Focus

A clear, broadly accepted vision is required to guide the process, and garner sustained support from diverse stakeholders (eg, “eHealth facilitated health care by 2020”; “Integrated eHealth-care in 5 years”).

### Principle 7: Provide Medium-Term Targets

Enunciating a specific goal that people can embrace helps build and maintain momentum, for example, “To establish a needs-based, evidence informed, and national 5-year eHealth strategic plan that adopts technologically appropriate and culturally sensitive eHealth solutions and guides eHealth policy development”.

## Process of eHealth Strategy Development

Developed originally as a Telehealth Strategy Development Framework [15], this tool has been adapted for eHealth and further enhanced by embedding strategy and cognitive process theory and approaches (described above). Identification of specific methods and processes for collecting, managing, and using the information gathered during implementation of the tool continues. Assuming the above seven principles have been embraced and employed, there are seven steps to development of an eHealth strategy (the 8^th^ step), which then guides and informs further undertakings, including the 9^th^ step (policy development), and subsequent steps, eg, design of an enterprise architecture plan, business plan, readiness assessment plan, implementation and change management plan, operational plan, evaluation plan, and so forth. The seven steps are described below.

### Evidence Gathering and Situation Assessment (Step 1)

To be effective, the eHealth strategy must address those specific health issues of most importance to the entity developing its eHealth strategy. Information regarding this will already be available in country/institution, NGO, or international agency reports (eg, WHO’s annual country health status reports), local or regional planning documents, administrative databases, as well as through literature review. The available information can be interrogated to reveal insight regarding what the issues are; what the causes and/or contributing factors are to each issue; how serious (size, scope) each issue is; who is impacted by each issue and where they are located; how many are impacted by each issue; what community/population characteristics may be related to each issue; what has been done in the past to address each issue; and why the interventions succeeded or failed.

In this way, the reports/literature (the evidence) will have identified the specific health issues (the needs) that must be addressed and allowed some analysis of any linkages between sociodemographic features, and health indicators, health risks, and service use. The process may also have revealed information gaps that may require addressing. This evidence gathering and situation assessment step establishes a sound foundation and baseline that is defensible to critics and also provides a preliminary list of areas where an eHealth application *may* offer a solution.

### Holistic Review (Step 2)

#### Overview

At this point, holistic situation assessment begins. It is necessary to examine many factors beyond just health needs to see if they guide decisions in a certain direction or identify potential barriers to some presumed solutions (this holistic approach has been used in other settings [40]). The goal is to examine the broader socioeconomic, political, and environmental context in relation to their impact on health need and to identify available assets, strengths, and capacity that might be brought to bear on the identified issues.

Although not an exhaustive list, information regarding each of the following examples will impact eHealth-related decisions.

#### Poverty (Spatial Distribution)

eHealth is considered by some as a tool for increasing equity of access to health care; mapping where poor and other vulnerable populations are located in relation to available health care facilities aids understanding of where implementation of eHealth solutions may be most beneficial [41].

#### Economic Policy Framework

ICT innovation continues to evolve, with new applications impacting all aspects of our economies and societies. Some reports suggest the public sector can begin large, expensive ICT projects without a clear understanding of goals, required resources, or risks. Understanding if and how government investment and policy formulation impacts ICT innovation, including eHealth, is essential [42].

#### Physical Geography

eHealth is recognized to remove or at least mitigate the barriers of time and space that physical geography imposes; however, technological solutions may also be limited by geography. Thus, solutions suitable for open expanses (eg, coastal areas, deserts) may not be suitable for extremely mountainous areas, highlighting the potential need for investment in different communication and technological eHealth solutions in different regions [43]. Consequently issues such as “line of sight” solutions, practical limits to wireless connectivity without repeater sites, location of infrastructure in disaster prone areas, etc, may restrict options.

#### Governance Issues and Policy Stability

Available experience with e-government, and the strength of the local supportive setting for eHealth will influence the acceptability and implementation of eHealth solutions. Furthermore, long-term vision, planning, and continuity in implementation despite political change is critical to success [30].

#### Cultural Barriers

Culture influences health care in several ways, including preferences for different treatments, individual health beliefs, and attitudes toward disclosure of medical information; eHealth solutions must be culturally appropriate if they are to be adopted and sustained [44,30].

#### Geopolitics

Factors such as geography, economics, and demography influence the politics, especially the foreign policy, of a country, which can influence intra- and interjurisdictional eHealth (eg, could neighboring countries support/share eHealth infrastructure or initiatives?)

#### Resource Issues (Including Human Health Resources)

Availability and skill set of the current cadre of health care (and eHealth) providers must be built to a critical point if countries are to “build the capacity to build their own capacity” [45].

#### e-Readiness or eHealth Readiness

Readiness to succeed in adoption, implementation, and use of any technology solutions is critical. The same is so for eHealth and the level of readiness of the public, health care providers, and the government must be thoroughly assessed (reassessed) to reveal gaps requiring intervention [46].

#### Linkages

NGOs or other agencies have become indispensable in the delivery of health in many developing countries [47]. However, these entities (eg, International Development Research Centre (IDRC), Swedish International Development Cooperation Agency (SIDA), Canadian International Development Agency (CIDA), International Red Cross, United States Agency for International Development (USAID), and faith-based organizations, etc) each have their own mandates, including perhaps eHealth strategies and activities. Without coordination and linkage of their activities to a specific eHealth strategy, their activities are, at best, ad hoc and confusing, and at worst, counterproductive, even detrimental.

#### Infrastructure

Fulmer [48] described infrastructure as the physical components of interrelated systems providing commodities and services essential to enable, sustain, or enhance societal living conditions. Given that eHealth is an ICT-based solution, the availability, type, capacity, coverage, cost, and location of current and planned physical ICT infrastructure will significantly influence the type and sophistication of eHealth solution feasible. Conversely, an eHealth Strategy may also inform discussion around just what type, capacity, coverage, cost, and location of planned infrastructure is needed.

#### Infostructure

In contrast to the “physical components”, infostructure can be described as those human resources, organizational and administrative structures, policies, regulations, and incentives that facilitate fully integrated and sustainable use of innovative ICTs and services to improve health care in an organized response to health and health care needs, issues, and challenges (ie, eHealth). Once again, completing an eHealth Strategy will also inform discussion around each of these issues.

The holistic review must also consider distant or unpredictable events (eg, climate change, humanitarian disasters, natural disasters), so that implemented solutions have sustainability and flexibility. Climate change remains debated, but whether natural or iatrogenic, some locations are predicted to turn from lakes into deserts or erase low-lying islands or traditional residential regions in estuaries, which will impact population movement and perhaps distribution of diseases. Humanitarian disasters (drought, war) can cause mass migration of populations across borders, stretching still further already stretched health care systems. Finally, natural disasters such as floods or earthquakes can cause extensive damage or destroy ICT infrastructure, and such considerations should impact the type and location of ICT infrastructure adopted during the strategy development process.

Individually and collectively, each of these factors has a bearing on the type of solution (and eHealth solution) that might be most appropriate and most sustainable for any identified health need and population. “Mind mapping” software (which creates diagrams of relationships between concepts, ideas or other pieces of information) is a valuable tool to assist in this process.

This holistic situation assessment step is crucial but is not typically or overtly undertaken. Absence of such a sound review undermines the credibility of any subsequent eHealth strategy, questions fiscal responsibility, and will adversely affect the sustainability of proposed applications.

### Differential Diagnosis (Step 3)

This is a tool taught to physicians during their training: “a systematic method of diagnosing a disorder that explains presenting signs and symptoms of a patient”. But what happens if 2 patients appear with similar signs and symptoms? Do they have the same disease? Maybe, but not necessarily. The signs and symptoms of 2 or more patients may be similar, but the actual diagnosis can be different and differential diagnosis allows this to be resolved. Because the diagnoses for the 2 patients are different, so too will be the *treatment* and management for each patient. This is analogous to assessing the health needs of different institutions, subnational regions within a country, or countries. The health *issues* and *settings* may be similar, but when examined carefully (holistic situation assessment), the real health needs (and possible eHealth solutions) are seen to be different. Sachs [40] applied this differential diagnosis approach to his economic assessment, and the same principle is applied here to differentiate possible solutions.

Using the data, information, and analysis garnered in Steps 1 and 2, it is possible to look at groupings at the next level down (eg, subnational entities for a national eHealth strategy, districts/wards for a regional eHealth strategy, and communities for an institutional eHealth strategy) and to reveal differing needs of distinct locales or populations. These should be highlighted for later consideration.

### Preliminary Prioritization (Step 4)

Given resource limitations, not every option can be pursued; trade-offs are essential and enforcing choice purposefully limits the options. But how do you choose? Priorities in health needs are traditionally viewed only in terms of disease morbidity and mortality. While intended for setting research priorities, the explicit and rational approach of the Combined Approach Matrix [49,50] takes into account other relevant determinants and can be used to prioritize the identified health needs. It consists of 5 different sources of evidence to formulate a priority list. These sources are (1) disease burden, (2) determinants, (3) level of knowledge, (4) economic cost, and (5) resources. Alternative, and perhaps more objective, tools are available (Sum of Ranking Approach (SRA), and Product of Value Approach (PVA)) as applied by the Ministry of Health and Long-Term Care (MOHLTC) in Ontario, Canada [51]. However, availability of sufficient quantity and quality of data for each health need may be a challenge. The overall goal of this step is to determine priority health needs and their associated characteristics for further review.

### Identifying Solutions (Step 5)

At this point in the process, the evidence-informed, and needs-based, health issues of the institution, region, or country are known and have been prioritized. In addition, the internal and external influences that the current and future setting may bring to bear are also understood. It is now possible to consider a variety of solutions to address these identified health issues. But it is essential that expansive thought be employed. These solutions need *not* involve technological intervention and might function at one or more of the practice, process, or policy levels.

This stage is the point at which to engage a broad selection of local (institution, region, or country) stakeholders, including government, private sector, and academic participants with diverse experience and expertise in health, education, and business to become an eHealth strategy advisory team. The group must be briefed using the material gathered and analyzed in Steps 1-4, thereby creating a well-informed and up-to-date team. Their task is to assess the identified and prioritized health needs, consider the political context, leverage existing (or recommend potential) partnerships, and develop innovative solutions to the top 20% of the prioritized health needs (note that innovation is often considered synonymous with the use of sophisticated technological solutions—this is *not* the case; a dictionary definition of innovation is simply something that is “new to you”). A secondary, but crucial, purpose of establishing this team is to begin the process of intrajurisdictional capacity building and developing a knowledgeable eHealth strategy culture.

### Considering eHealth Solutions (Step 6)

Only at this stage is the possible application of eHealth interventions considered. This process is best undertaken with the assistance of local or (if insufficient in number or expertise) external eHealth experts (telehealth, health informatics, e-learning, and e-commerce), who then become a part of the local working group and are briefed on both the prior material (Steps 1-4) and the process and solutions identified in Step 5. Again, expansive thinking is essential; many eHealth solutions are available but each may be optimal for only specific settings. It is recommended that attention still be focused on the top 20%; eHealth solutions may well be feasible for the remaining 80%, but if they are not highly prioritized then funding such initiatives may not be the wisest investment.

Options must be limited to a small number, and for each proposed eHealth application a brief but structured review (essentially a summary “business case”) must be prepared. This will help to assess the feasibility of each solution for the given institution, region, or country; not all of the proposed eHealth applications will be technologically appropriate, culturally sensitive, or financially feasible.

### Secondary Prioritization (Step 7)

Almost invariably more than one eHealth solution is available for any specific need (eg, applying different technologies such as videoconferencing versus podcasts for CME of clinicians), and more than one need can be addressed using eHealth (eg, telehealth consultation services to remote communities versus introduction of a public health surveillance tool). Decisions must be made. The business case analysis will have provided insight regarding potential cost, complexity of implementation, likely readiness to implement, and proportion of the population impacted—these features can be used to rank options as described earlier. In the absence of sufficient data to permit objective prioritization, then a more subjective approach will have to be taken. Here, each potential solution can be classified into applications that are considered *essential* to have, versus those that would be *good* to have, versus those that might be *nice* to have. eHealth solutions that address a high priority health need of modest or low cost and complexity, and impact a large proportion of the population would be optimal and identified as essential. This is a critical stage in the eHealth strategy development process, as it sets direction for allocation of resources and commits to a certain path of ICT infrastructure development and policy need.

### Strategy Formulation (Step 8)

To create the institution, region, or country “eHealth strategy”, the findings from Steps 1-7 are synthesized, and the recommended priority needs and selected eHealth solutions described. This eHealth strategy document then informs further action. It will guide the building of the necessary enterprise architecture, ICT infrastructure, processes, and policy environment, as well as the subsequent design, readiness assessment, implementation plan, change management plan, evaluation study, and sustainability program for the selected eHealth applications.

### Policy Development

There is continued debate about strategy versus policy. While there may be no clear cut answer to “what comes first—the chicken or the egg?”, logically it follows that poor inputs to designing an eHealth favorable policy environment will ultimately result in poor outputs, poor outcomes, and undesirable impacts. Consequently, this paper is intended to encourage development of a sound and evidence-based eHealth strategy for any entity (region, country, subnational jurisdiction, or health care facility) as the *first* step: the premise being that strategy defines *where* and *why* action should be taken, whereas policy describes and implements *how* that action should be taken. During the strategy development process described above, barriers and facilitators to implementing the planned eHealth strategy will have been identified and documented highlighting specific areas of policy need in an evidence-based fashion.

Approaching eHealth policy development in this way ensures that important issues requiring policy solutions (new, revised, or rescinded policy) are identified. Attention can then be given to considering what specific eHealth policy is required to encourage and or manage the strategy, including expected growth in implementation and evaluation of eHealth solutions and sustainability. Such policy must be developed through an iterative, collaborative, and participatory process if support is to be engendered from all stakeholders. Further, it must be remembered that eHealth specific policy is only developed where it is not possible to achieve the desired result through revision/amendment of existing health, education, or ICT policy. In the end, eHealth should become just another tool by which to provide health-related information, education, and services—to do so it must become integral to the existing health care system, not separate from it.

## Discussion

### eHealth Strategy Development

In our culture of constant growth, more and more governments and decision makers (eg, senior managers of health care facilities) are being called to task to demonstrate the value of the decisions they make. Health seemingly consumes a greater and greater proportion of available funds in an attempt to address the complex health care issues that plague all countries as we continue the global transition from infectious to noninfectious and chronic disease and old age. eHealth—a relatively new approach available to decision and policy makers in the developing world—has been hailed by many as a solution to these woes. Yet attempts to date in the developed world have shown relatively little success or return on investment despite significant outlay. A better and more reasoned means of understanding where and how to apply eHealth solutions is necessary. An evidence- and needs-based, transparent, and defensible eHealth strategy is required by each region, country, and facility.

Only recently has some guidance for eHealth strategy development become available. In 2011, the Commonwealth provided many templates and a structure to use in initiating a series of workshops intended to lead towards development of an eHealth strategy [14]. In that document, a good job was done of encouraging broad understanding of eHealth options as potential solutions (eg, Table 11 in [14]) and projecting future costs (eg, Table 12 in [14]). However, the toolkit does not describe processes for holistic situation assessment nor for prioritization of both health needs and eHealth solutions. More recently, the WHO/ITU provided a comprehensive document (a National eHealth Strategy Toolkit) [6]. Although promoted as a tool by which to create eHealth strategy, the content does not deliver a strategy per se, but rather provides guidance to achieve three outputs: (1) a National eHealth Vision, (2) a National eHealth Action Plan, and (3) Monitoring and Evaluation processes. Indeed, the document does not clearly distinguish between vision and strategy, nor seemingly identify development of an eHealth strategy as a specific output. It is certainly a comprehensive and valuable document, with much guidance provided on many steps to be taken after eHealth strategy development is complete.

While both these documents contribute significantly to the debate, neither provides conceptual background or theoretical support to the need for, and development of, eHealth strategy, nor do they focus on development of an eHealth strategy as a distinct and primary undertaking. Instead, both intermingle many other issues (eg, interoperability, standards, confidentiality, security, policy, architecture, implementation, change management, investment, benefits realization), which serves only to distract from the primary intended goal of eHealth strategy development. All of these aforementioned topics are certainly of relevance but should be addressed only once a clear understanding has been developed of “where you want to go” and “why you want to go there”, that is, having established the eHealth strategy.

Perhaps the greatest contribution of the eHealth Strategy Development Framework is its clear focus on establishing an evidence-based eHealth strategy and providing the conceptual understanding and tools required by which to achieve that. To be effective, an eHealth strategy must be solidly grounded in an understanding of the broader context within the setting (region, country, facility), and the challenges and opportunities that exist. It must provide clarity around the health need(s) that must be addressed and the solutions (particularly eHealth solutions) it is intended to apply.

The eHealth Strategy should not be so detailed and unwieldy that it cannot be used as a functional and guiding document. Therefore, it does not provide details of specific undertakings; those needs are addressed through next steps, including design of an enterprise architecture plan, business plan, readiness assessment plan, implementation plan, change management plan, evaluation plan, and operational plan. Once established, the eHealth Strategy acts as a pole star, that is, a “constant” to which all can refer as they work to achieve identified goals and navigate the defined path.

### Conclusion

Growing expectations, changing demographics and disease patterns, and resource limitations require wise investment in eHealth solutions that address major health needs in any given setting. Solutions that are designed and implemented now must form the foundation (practice and technology infrastructure) for decades to come. Such sustainable eHealth solutions first require development of a sound, evidence-based, transparent, and defensible eHealth strategy, which then informs subsequent development of a sound and viable policy environment, enterprise architecture, and so forth. This paper describes the conceptual understanding and practical steps required for any facility, country, or region to develop its own eHealth strategy.

## Abbreviations

CIDA: Canadian International Development Agency
CME: continuing medical education
ECG: electrocardiograph
EU: European Union
GDP: gross domestic product
HIS: health information system
HIT: health information technology
ICT: information and communications technologies
IDRC: International Development Research Centre
IRC: International Red Cross
ITU: International Telecommunications Union
KT: knowledge transfer/translation
MOHLTC: Ministry of Health and Long-Term Care
NGO: non-governmental organization
NHS: National Health Service
OECD: Organization for Economic Co-operation and Development
PAHO: Pan American Health Organization
PVA: product of value approach
SER: socioeconomic return
SIDA: Swedish International Development Cooperation Agency
SRA: sum of ranking approach
USAID: United States Agency for International Development
WHO: World Health Organization
